# Supplementary material for: Tyrosinase Inhibitors Derived from Chemical Constituents of Dianella ensifolia
Source: Plants (Basel). 2022 Aug 18;11(16):2142. doi: 10.3390/plants11162142 (PMC9414913; doi:10.3390/plants11162142)
Supplement: Supplementary file 1 [file plants-11-02142-s001.zip › plants-1860944-supplementary.pdf]

Supplementary Materials:

Tyrosinase Inhibitors Derived from Chemical Constituents of *Dianella ensifolia*

Yu-Chang Chen [1], Sheng-Han Su [2,†], Jheng-Cian Huang [2,†], Che-Yi Chao [3], Ping-Jyun Sung [4], Yih-Fung Chen [5,6], Horng-Huey Ko [1,6,7,8,*] and Yueh-Hsiung Kuo [9,10,11,*]

[1] School of Pharmacy, College of Pharmacy, Kaohsiung Medical University, Kaohsiung 807, Taiwan
[2] Department of Chemistry, National Taiwan University, Taipei 106, Taiwan
[3] Department of Food Nutrition and Health Biotechnology, Asia University, Taichung 413, Taiwan
[4] National Museum of Marine Biology and Aquarium, Pingtung 944, Taiwan
[5] Graduate Institute of Natural Products, College of Pharmacy, Kaohsiung Medical University, Kaohsiung 807, Taiwan
[6] Department of Medical Research, Kaohsiung Medical University Hospital, Kaohsiung Medical University, Kaohsiung 807, Taiwan
[7] Department of Fragrance and Cosmetic Science, College of Pharmacy, Kaohsiung Medical University, Kaohsiung 807, Taiwan
[8] Drug Development and Value Creation Center, Kaohsiung Medical University, Kaohsiung 807, Taiwan
[9] Department of Chinese Pharmaceutical Sciences and Chinese Medicine Resources, College of Pharmacy, China Medical University, Taichung 404, Taiwan
[10] Chinese Medicine Research Center, China Medical University, Taichung 404, Taiwan
[11] Department of Biotechnology, Asia University, Taichung 413, Taiwan

* Correspondence: hhko@kmu.edu.tw (H.-H.K.); kuoyh@mail.cmu.edu.tw (Y.-H.K.); Tel.: +886-7-3121101 (ext. 2643) (H.-H.K.); +886-4-22053366 (ext. 5709) (Y.-H.K.)
† These authors contributed equally to this work.

Figure S1. The chemical structures of compounds **1–71**.
Figure S2. IR spectrum of (2*S*)-4'-hydroxy-6,7-dimethoxyflavan (**1**)
Figure S3. EIMS spectrum of (2*S*)-4'-hydroxy-6,7-dimethoxyflavan (**1**)
Figure S4. The ^{1}H-NMR spectrum of (2*S*)-4'-hydroxy-6,7-dimethoxyflavan (**1**; 400 MHz, $CDCl_3$)
Figure S5. The ^{13}C-NMR spectrum of (2*S*)-4'-hydroxy-6,7-dimethoxyflavan (**1**; 100 MHz, $CDCl_3$)
Figure S6. COSY spectrum of (2*S*)-4'-hydroxy-6,7-dimethoxyflavan (**1**)
Figure S7. NOESY spectrum of (2*S*)-4'-hydroxy-6,7-dimethoxyflavan (**1**)
Figure S8. HMQC spectrum of (2*S*)-4'-hydroxy-6,7-dimethoxyflavan (**1**)
Figure S9. HMBC spectrum of (2*S*)-4'-hydroxy-6,7-dimethoxyflavan (**1**)
Figure S10. IR spectrum of (2*S*)-3',4'-dihydroxy-7-methoxy-8-methylflavan (**2**)
Figure S11. EIMS spectrum of (2*S*)-3',4'-dihydroxy-7-methoxy-8-methylflavan (**2**)
Figure S12. The ^{1}H-NMR spectrum of (2*S*)-3',4'-dihydroxy-7-methoxy-8-methylflavan (**2**; 500 MHz, $CDCl_3$)
Figure S13. The ^{13}C-NMR spectrum of (2*S*)-3',4'-dihydroxy-7-methoxy-8-methylflavan (**2**; 125 MHz, $CDCl_3$)
Figure S14. COSY spectrum of (2*S*)-3',4'-dihydroxy-7-methoxy-8-methylflavan (**2**)
Figure S15. NOESY spectrum of (2*S*)-3',4'-dihydroxy-7-methoxy-8-methylflavan (**2**)
Figure S16. HMQC spectrum of (2*S*)-3',4'-dihydroxy-7-methoxy-8-methylflavan (**2**)
Figure S17. HMBC spectrum of (2*S*)-3',4'-dihydroxy-7-methoxy-8-methylflavan (**2**)
Figure S18. IR spectrum of (2*S*)-2'-hydroxy-7-methoxyflavan (**3**)
Figure S19. EIMS spectrum of (2*S*)-2'-hydroxy-7-methoxyflavan (**3**)
Figure S20. The ^{1}H-NMR spectrum of (2*S*)-2'-hydroxy-7-methoxyflavan (**3**; 500 MHz, $CDCl_3$)
Figure S21. The ^{13}C-NMR spectrum of (2*S*)-2'-hydroxy-7-methoxyflavan (**3**; 125 MHz, $CDCl_3$)
Figure S22. COSY spectrum of (2*S*)-2'-hydroxy-7-methoxyflavan (**3**)
Figure S23. NOESY spectrum of (2*S*)-2'-hydroxy-7-methoxyflavan (**3**)
Figure S24. HMQC spectrum of (2*S*)-2'-hydroxy-7-methoxyflavan (**3**)
Figure S25. HMBC spectrum of (2*S*)-2'-hydroxy-7-methoxyflavan (**3**)
Figure S26. IR spectrum of 4-hydroxy-4-(7-methoxy-8-methylchroman-2-yl)-cyclohex-2-enone (**4**)
Figure S27. EIMS spectrum of 4-hydroxy-4-(7-methoxy-8-methylchroman-2-yl)-cyclohex-2-enone (**4**)
Figure S28. The ^{1}H-NMR spectrum of 4-hydroxy-4-(7-methoxy-8-methylchroman-2-yl)-cyclohex-2-enone (**4**; 400 MHz, $CDCl_3$)
Figure S29. The ^{13}C-NMR spectrum of 4-hydroxy-4-(7-methoxy-8-methylchroman-2-yl)-cyclohex-2-enone (**4**; 100 MHz, $CDCl_3$)
Figure S30. COSY spectrum of 4-hydroxy-4-(7-methoxy-8-methylchroman-2-yl)-cyclohex-2-enone (**4**)
Figure S31. NOESY spectrum of 4-hydroxy-4-(7-methoxy-8-methylchroman-2-yl)-cyclohex-2-enone (**4**)
Figure S32. HMQC spectrum of 4-hydroxy-4-(7-methoxy-8-methylchroman-2-yl)-cyclohex-2-enone (**4**)
Figure S33. HMBC spectrum of 4-hydroxy-4-(7-methoxy-8-methylchroman-2-yl)-cyclohex-2-enone (**4**)

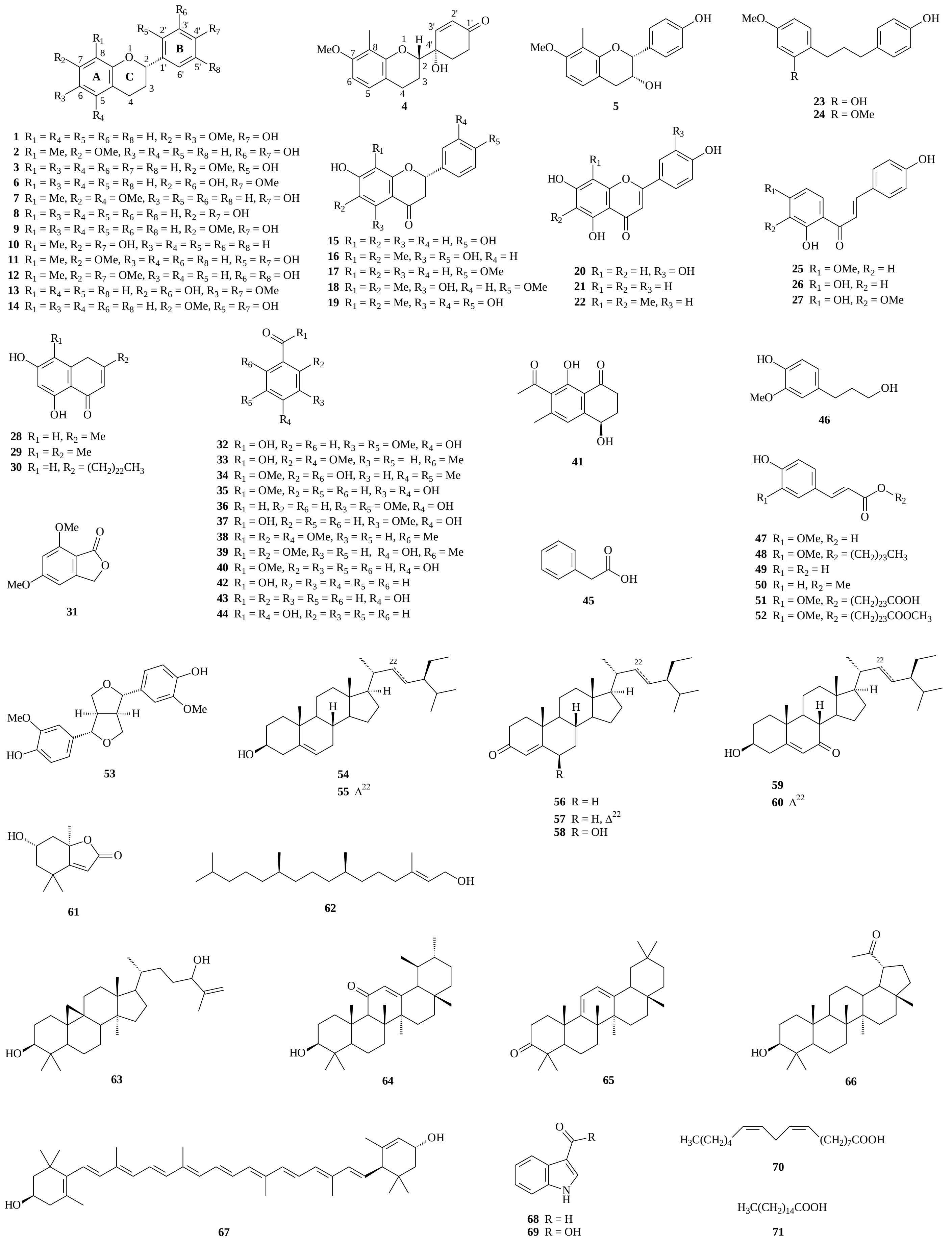

Figure S1. The chemical structures of compounds **1–71**.

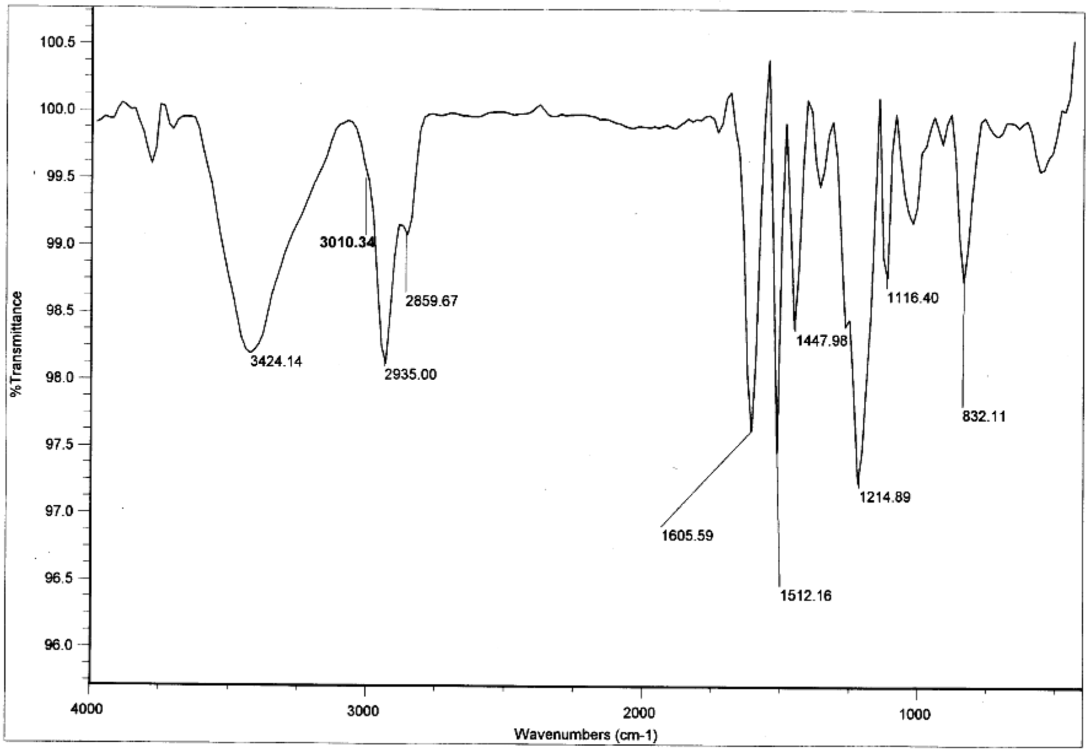

Figure S2. IR spectrum of (2*S*)-4'-hydroxy-6,7-dimethoxyflavan (**1**)

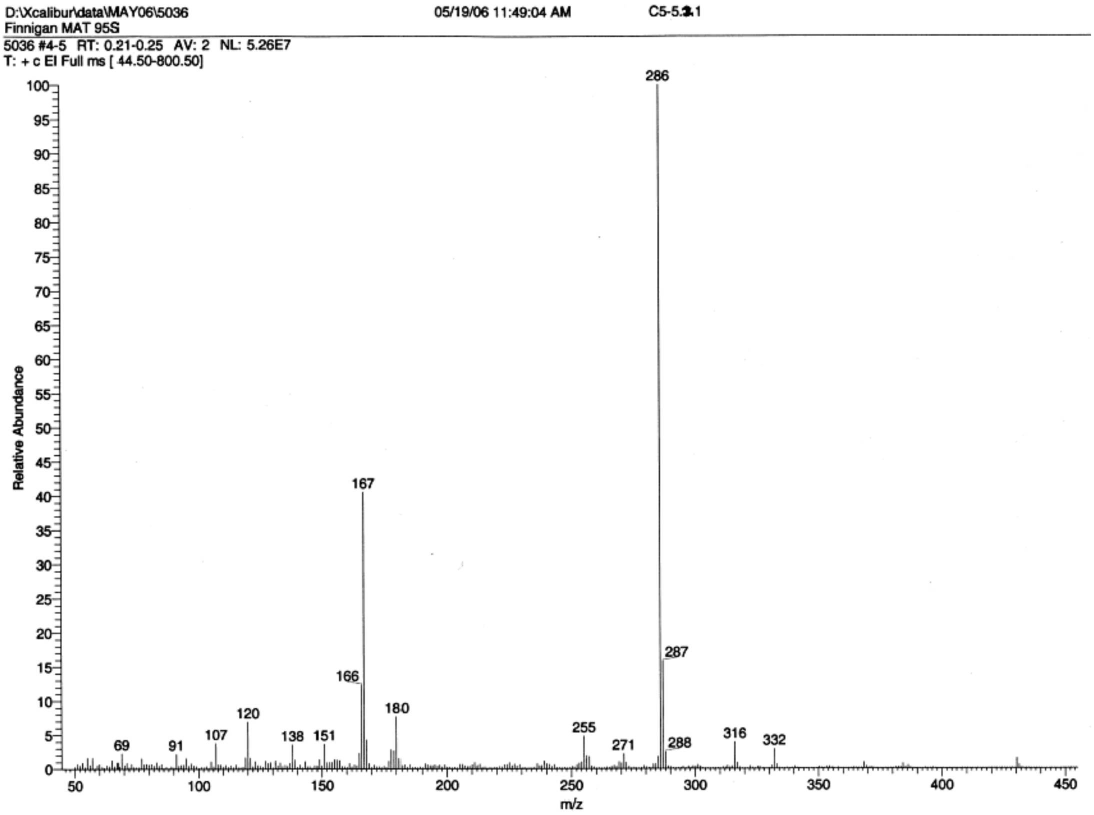

Figure S3. EIMS spectrum of (2*S*)-4'-hydroxy-6,7-dimethoxyflavan (**1**)

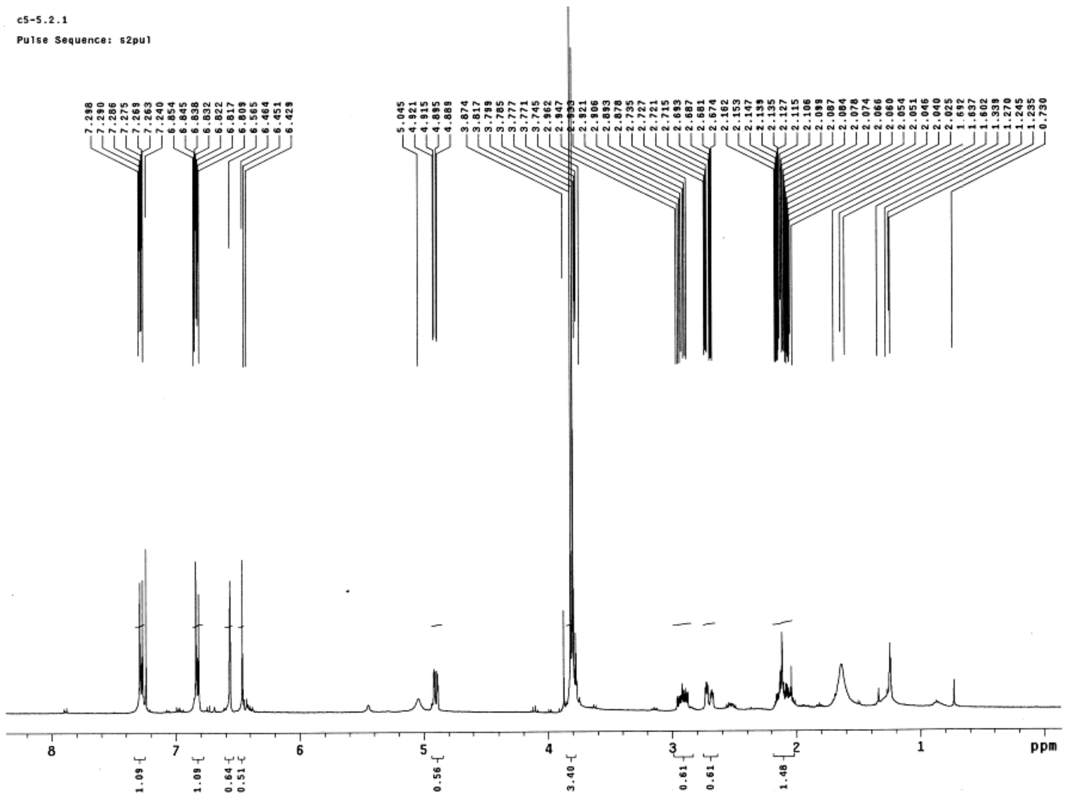

Figure S4. The ^{1}H-NMR spectrum of (2*S*)-4'-hydroxy-6,7-dimethoxyflavan (**1**; 400 MHz, $CDCl_3$)

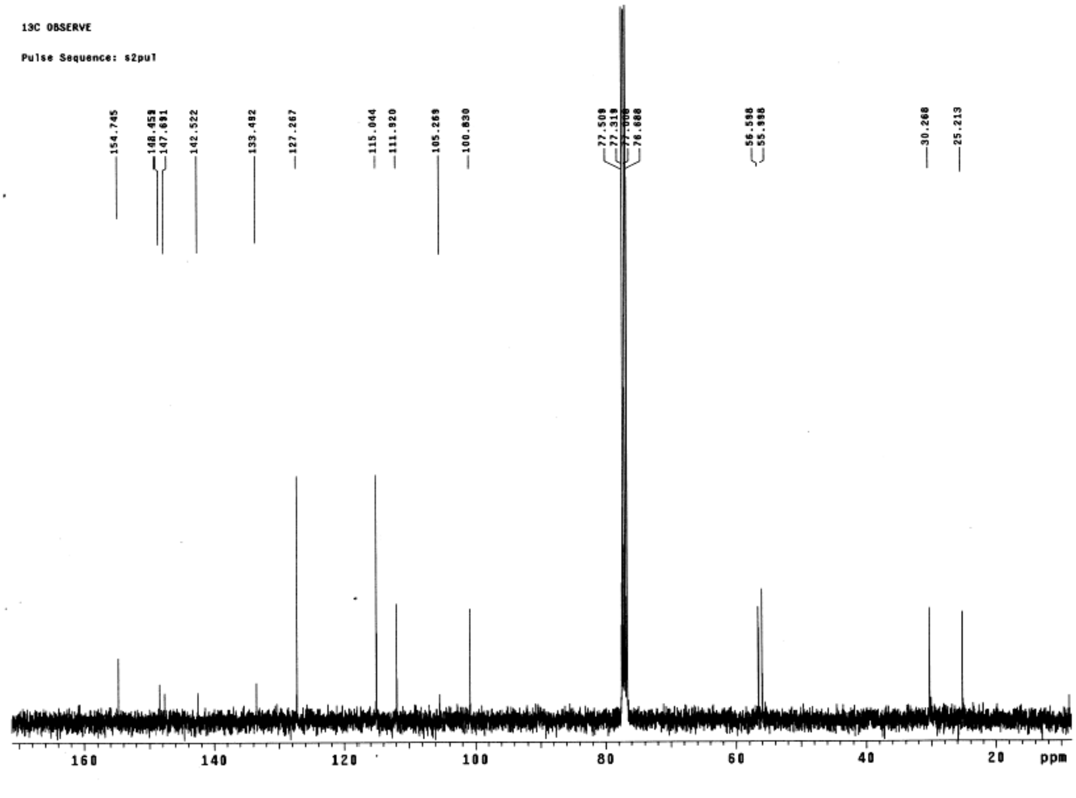

Figure S5. The ^{13}C-NMR spectrum of (2*S*)-4'-hydroxy-6,7-dimethoxyflavan (**1**; 100 MHz, $CDCl_3$)

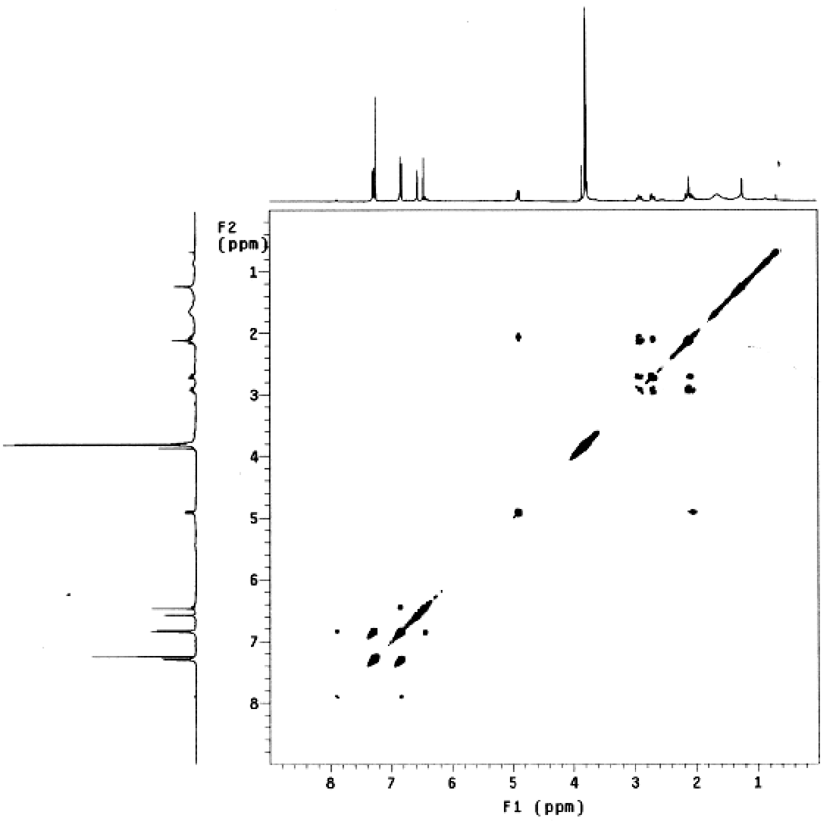

Figure S6. COSY spectrum of (2*S*)-4'-hydroxy-6,7-dimethoxyflavan (**1**)

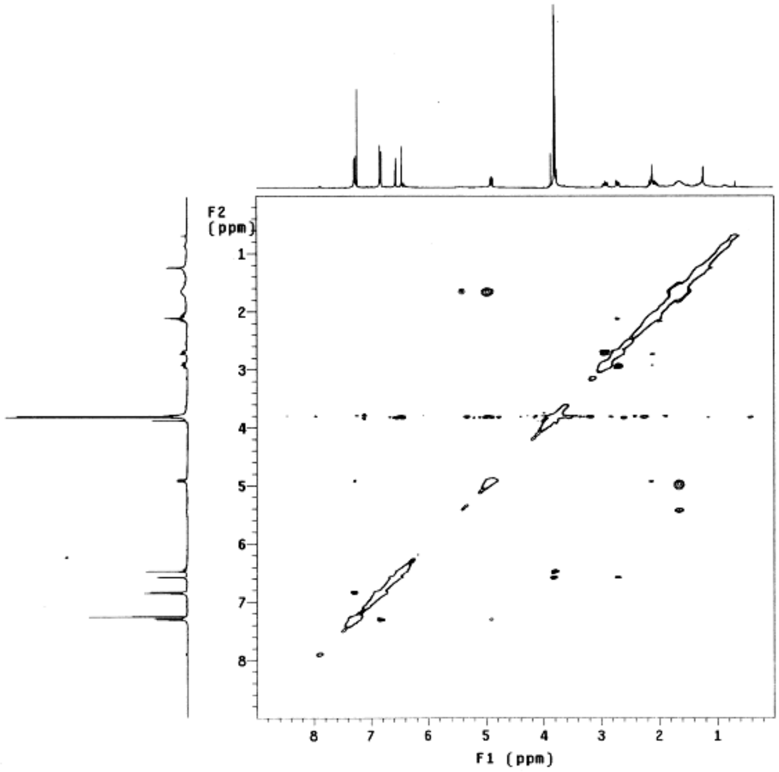

Figure S7. NOESY spectrum of (2*S*)-4'-hydroxy-6,7-dimethoxyflavan (**1**)

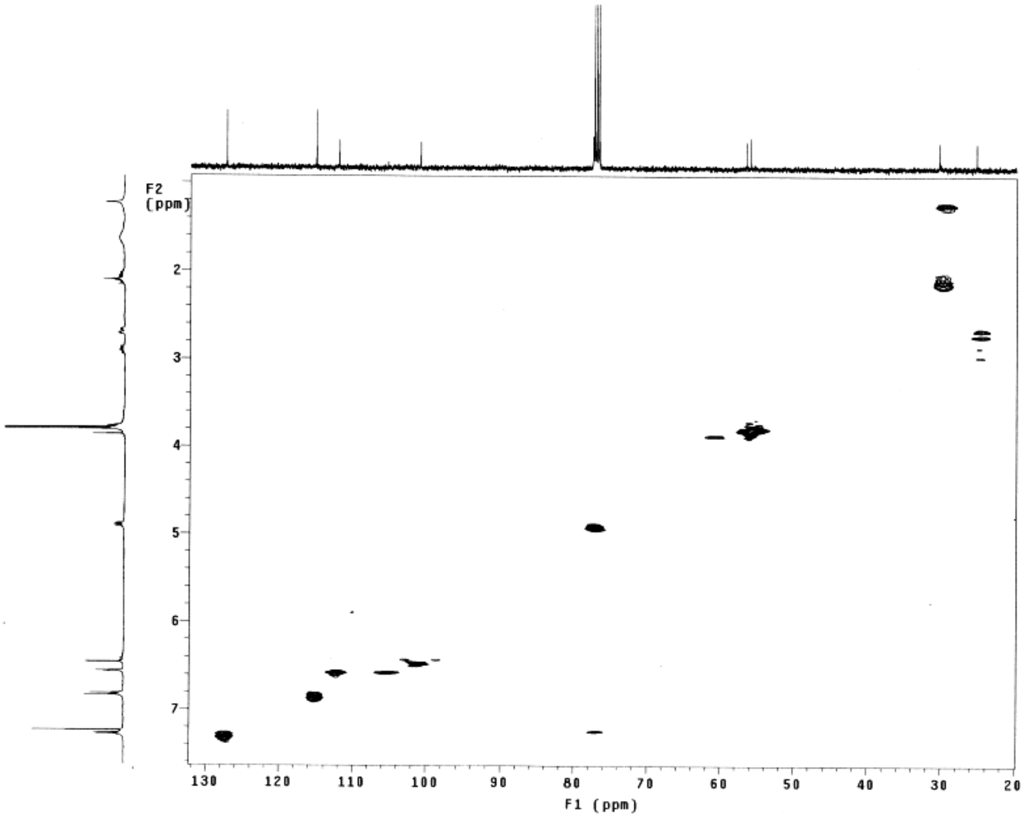

Figure S8. HMQC spectrum of (2*S*)-4'-hydroxy-6,7-dimethoxyflavan (**1**)

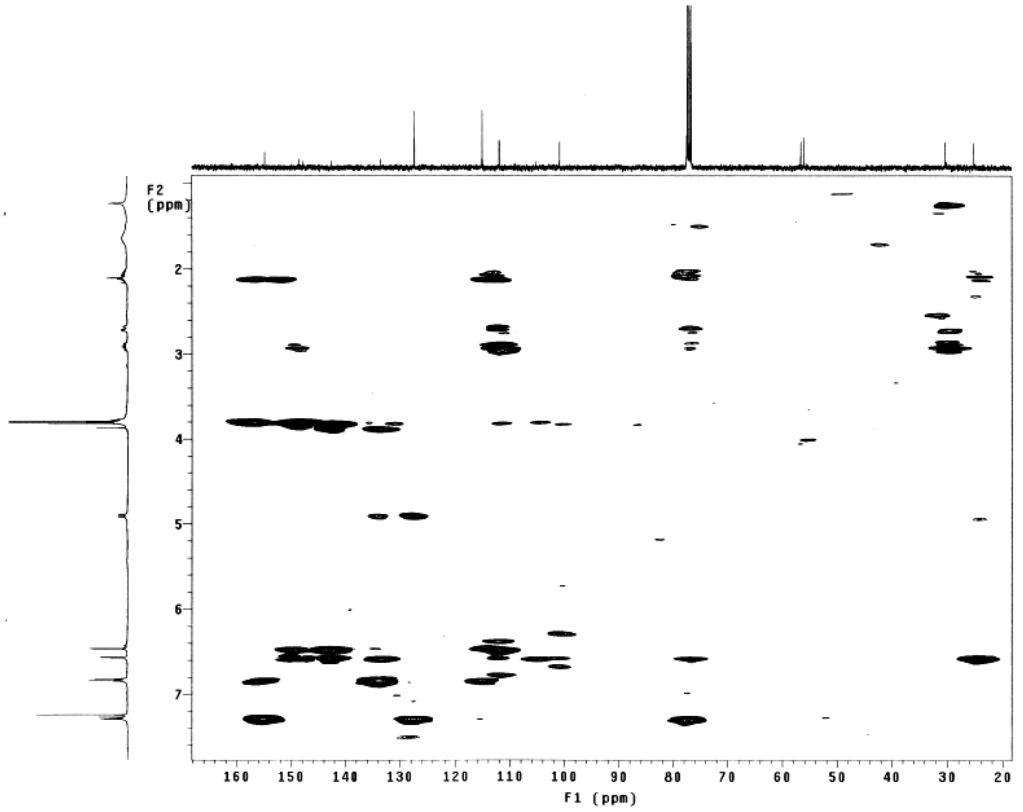

Figure S9. HMBC spectrum of (2*S*)-4'-hydroxy-6,7-dimethoxyflavan (**1**)

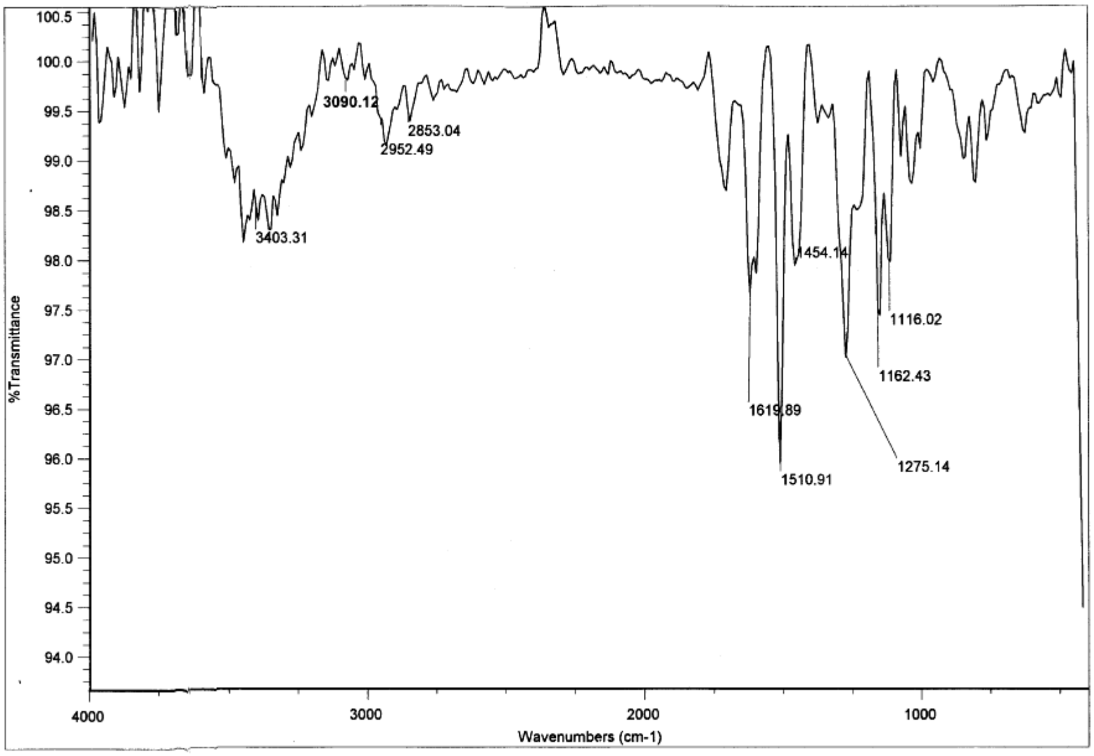

Figure S10. IR spectrum of (2*S*)-3',4'-dihydroxy-7-methoxy-8-methylflavan (**2**)

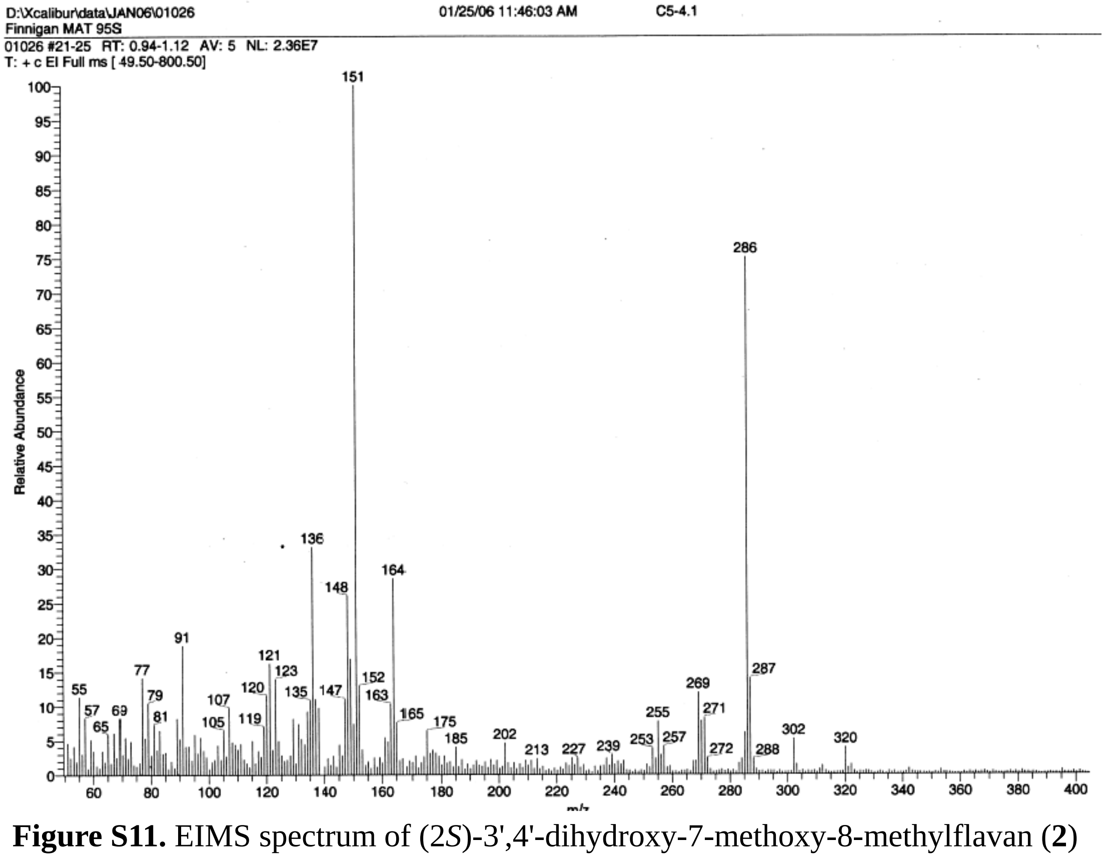

Figure S11. EIMS spectrum of (2*S*)-3',4'-dihydroxy-7-methoxy-8-methylflavan (**2**)

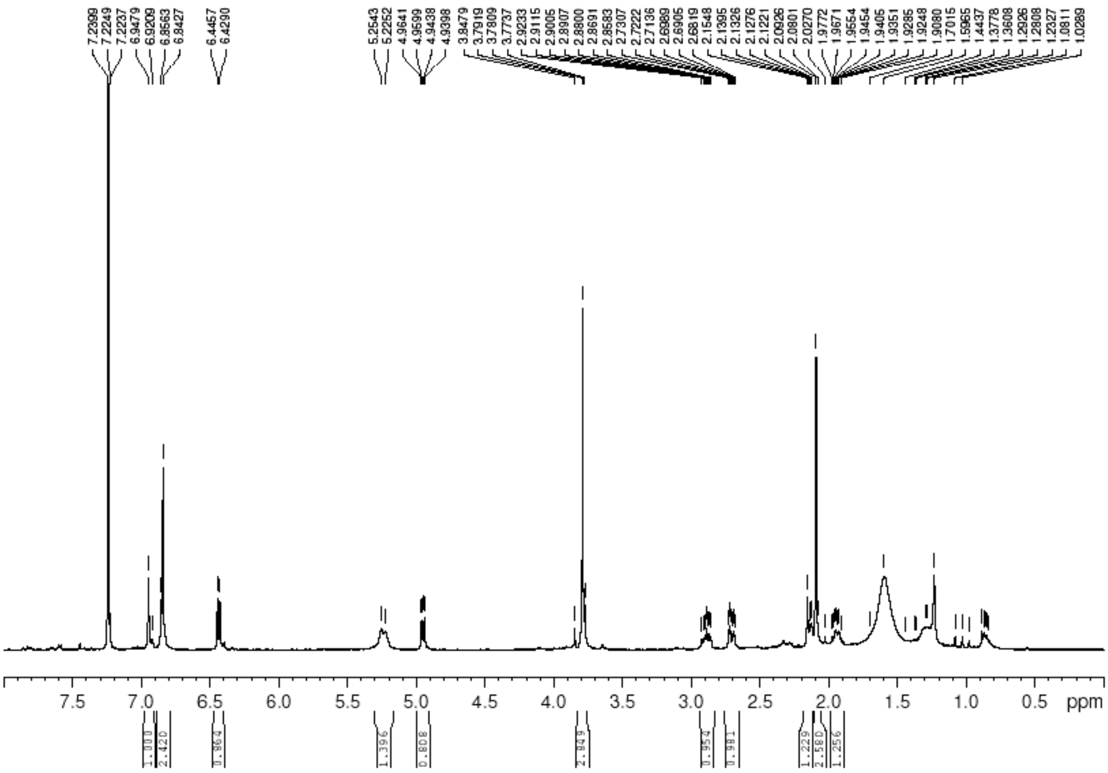

Figure S12. The ^{1}H-NMR spectrum of (2*S*)-3',4'-dihydroxy-7-methoxy-8-methylflavan (**2**; 500 MHz, $CDCl_3$)

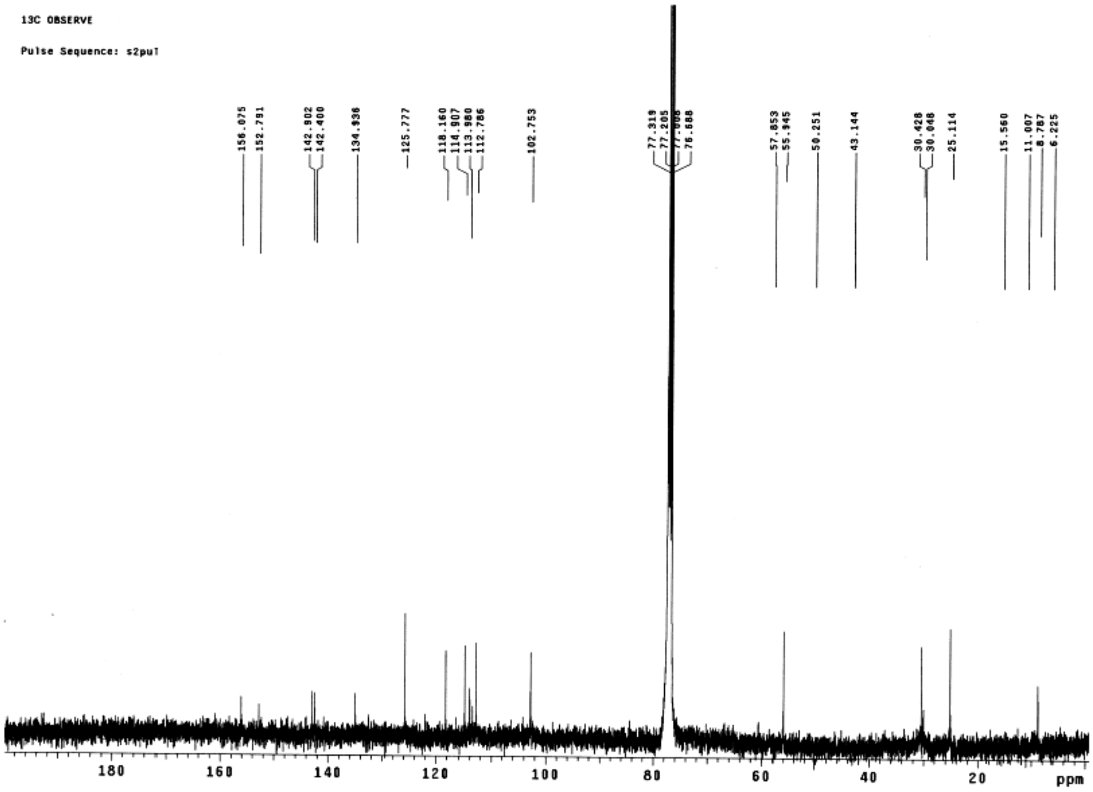

Figure S13. The ^{13}C-NMR spectrum of (2*S*)-3',4'-dihydroxy-7-methoxy-8-methylflavan (**2**; 125 MHz, $CDCl_3$)

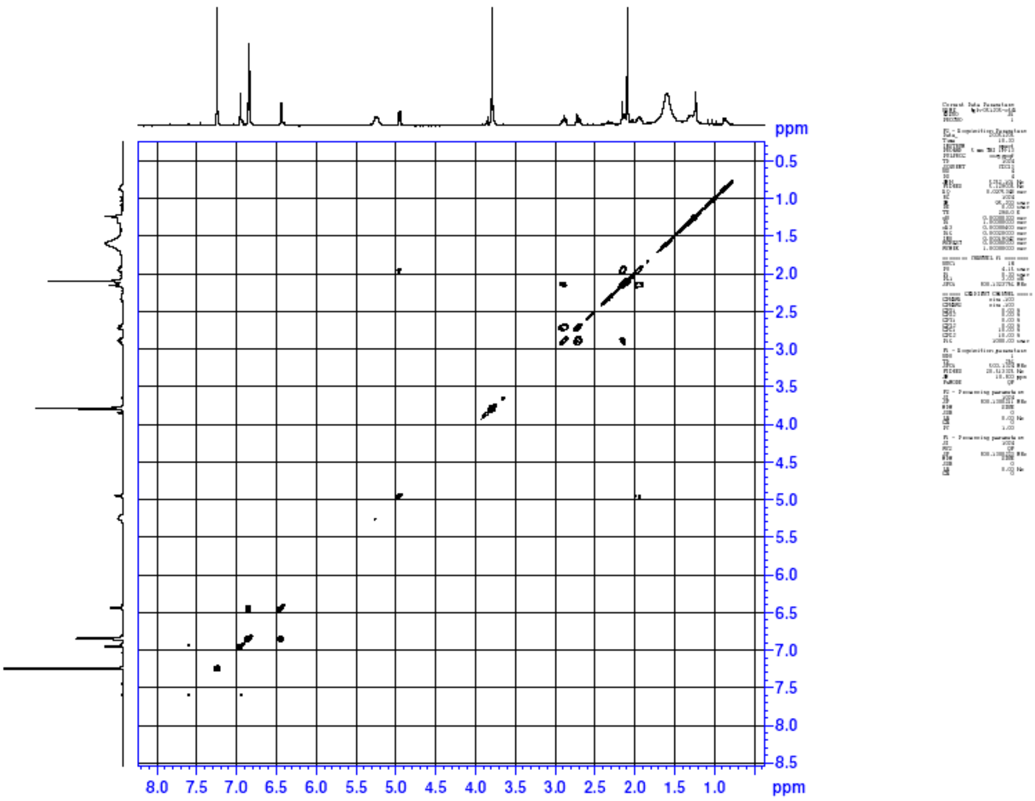

Figure S14. COSY spectrum of (2*S*)-3',4'-dihydroxy-7-methoxy-8-methylflavan (**2**)

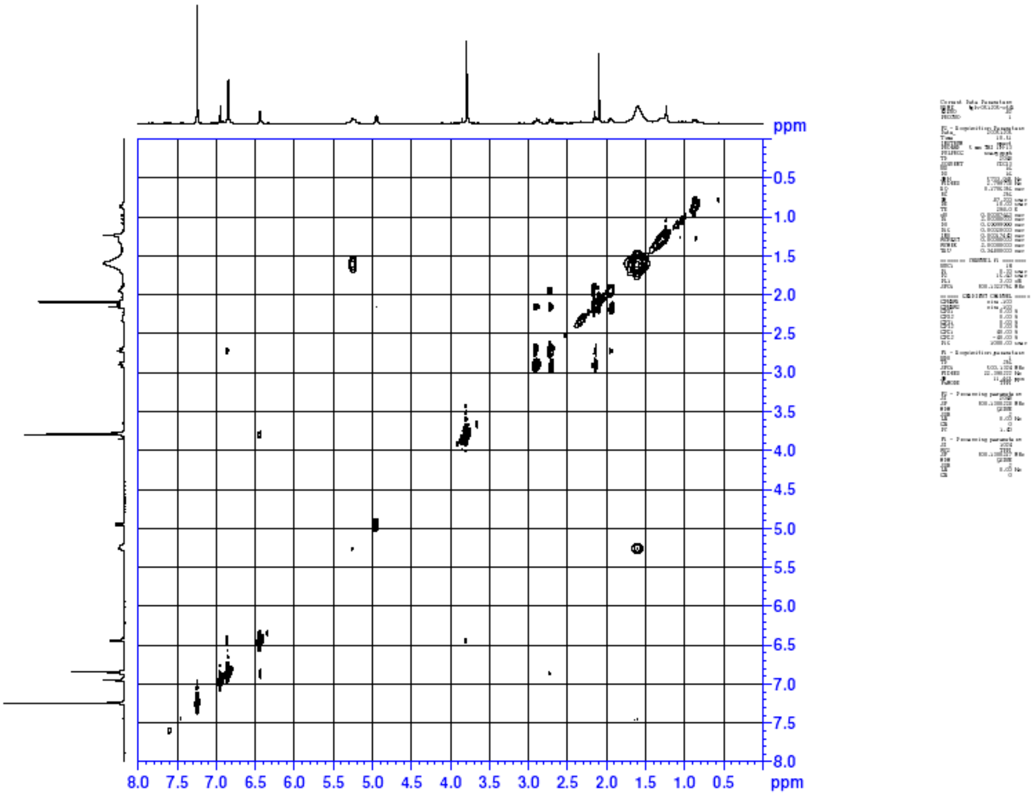

Figure S15. NOESY spectrum of (2*S*)-3',4'-dihydroxy-7-methoxy-8-methylflavan (**2**)

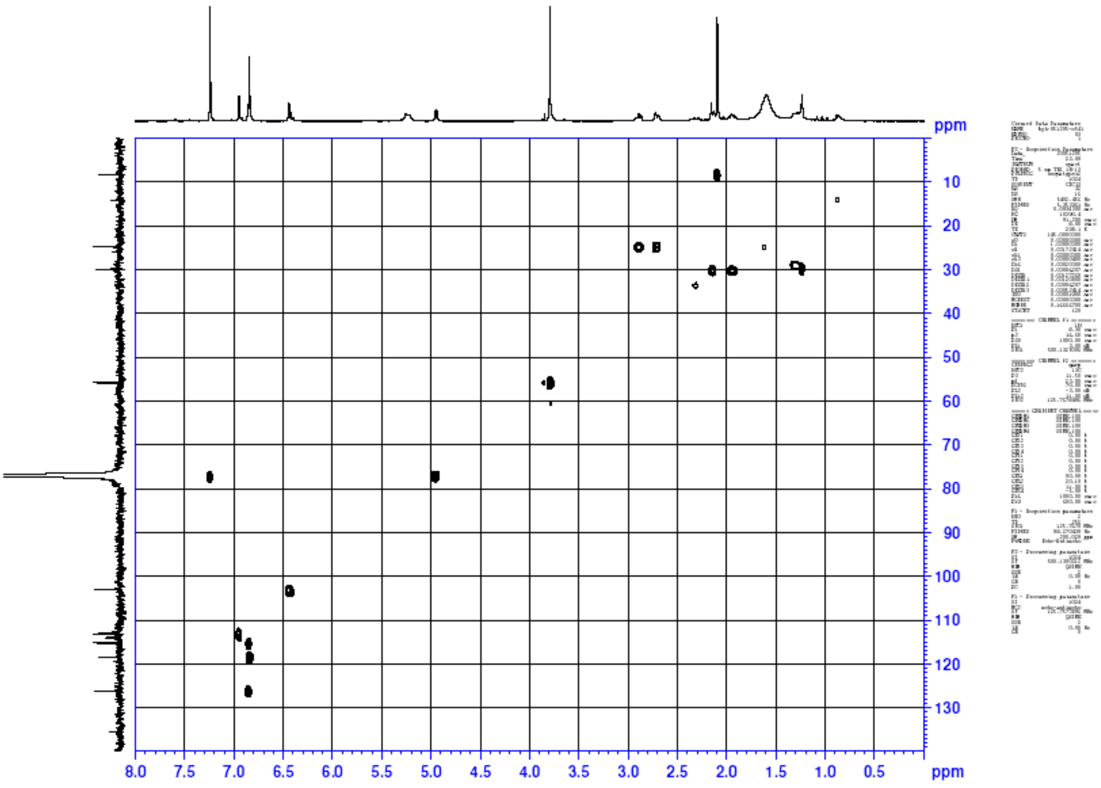

Figure S16. HMQC spectrum of (2*S*)-3',4'-dihydroxy-7-methoxy-8-methylflavan (**2**)

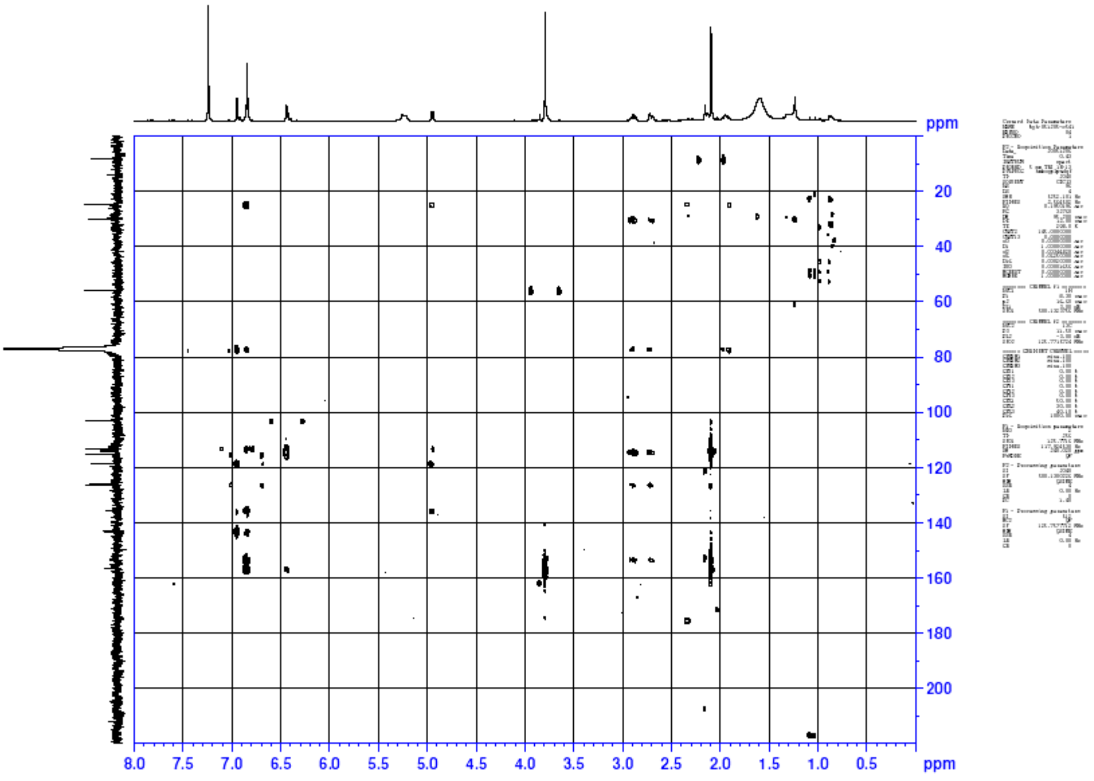

Figure S17. HMBC spectrum of (2*S*)-3',4'-dihydroxy-7-methoxy-8-methylflavan (**2**)

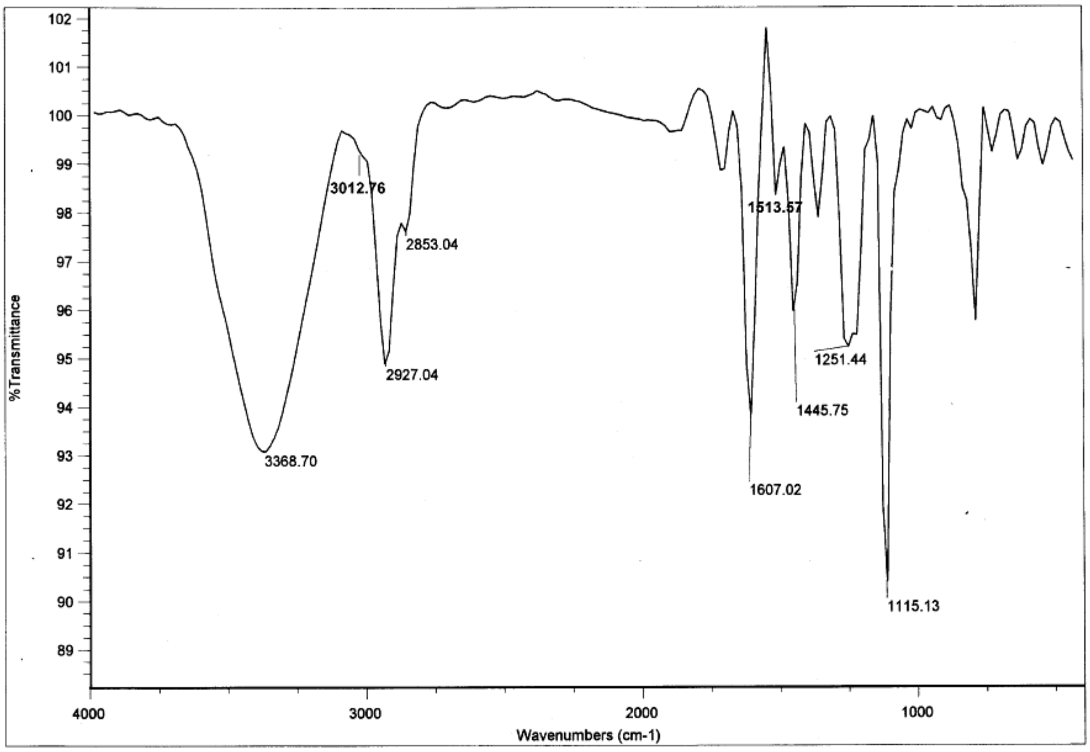

Figure S18. IR spectrum of (2*S*)-2'-hydroxy-7-methoxyflavan (**3**)

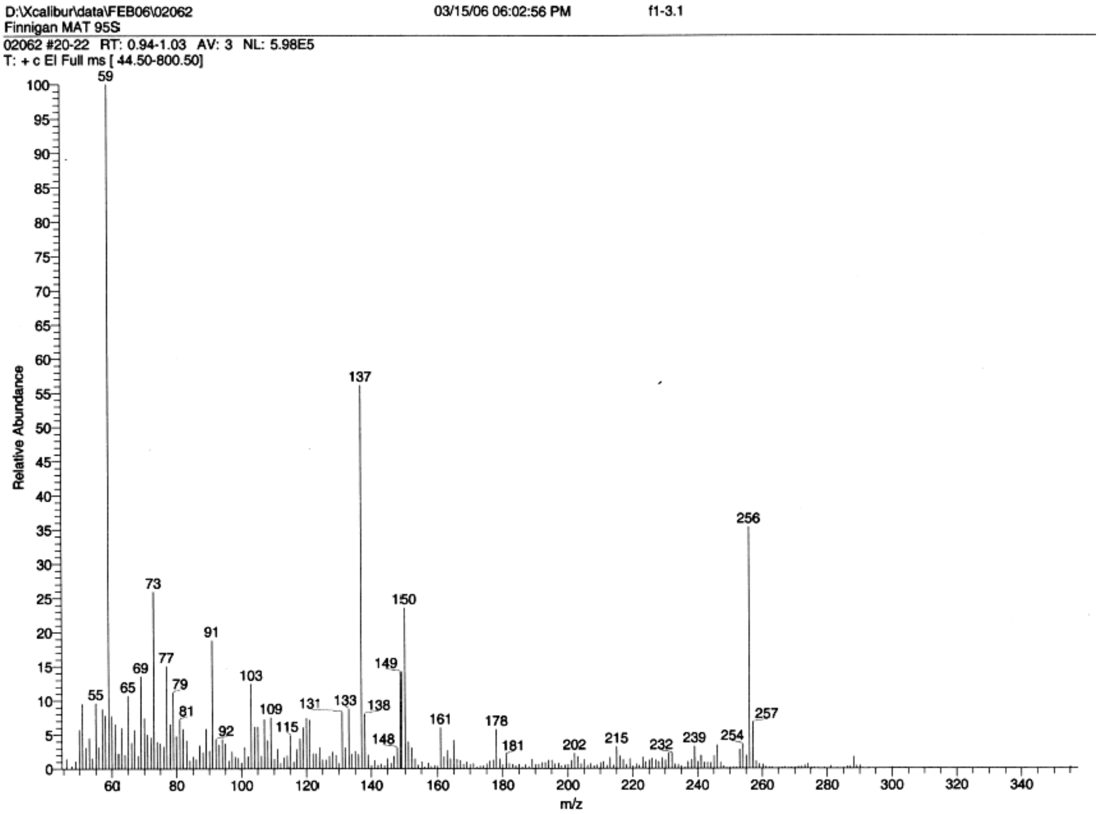

Figure S19. EIMS spectrum of (2*S*)-2'-hydroxy-7-methoxyflavan (**3**)

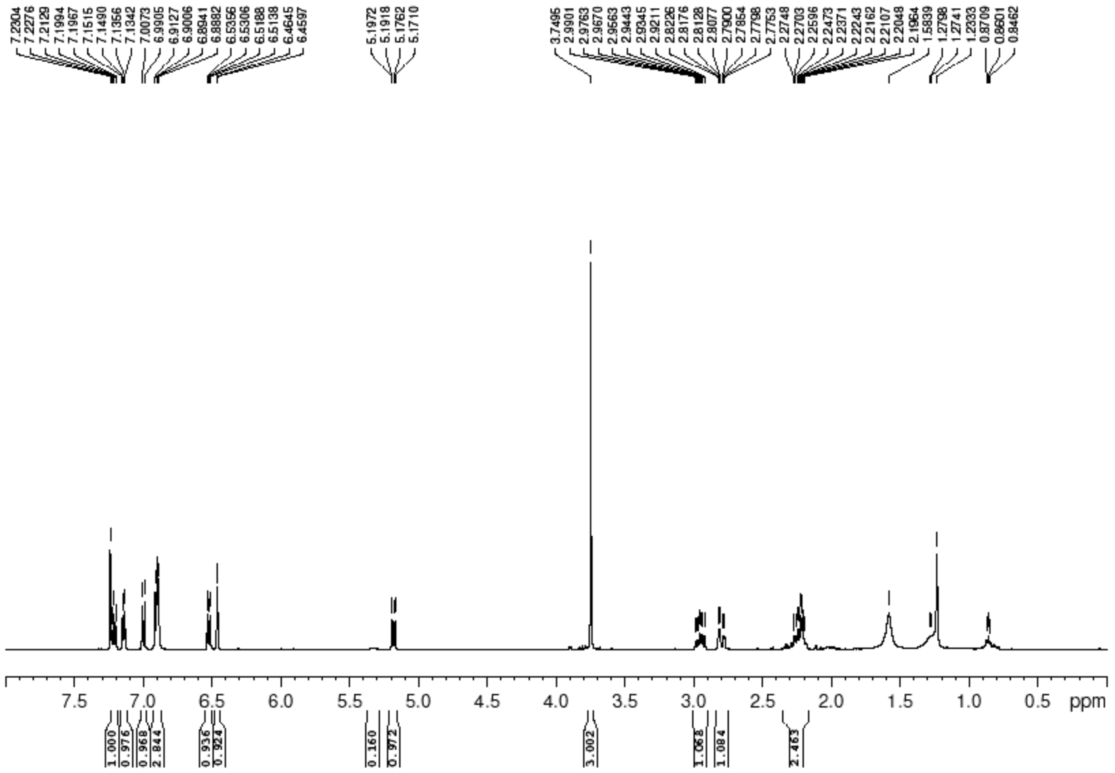

Figure S20. The ^{1}H-NMR spectrum of (2*S*)-2'-hydroxy-7-methoxyflavan (**3**; 500 MHz, $CDCl_3$)

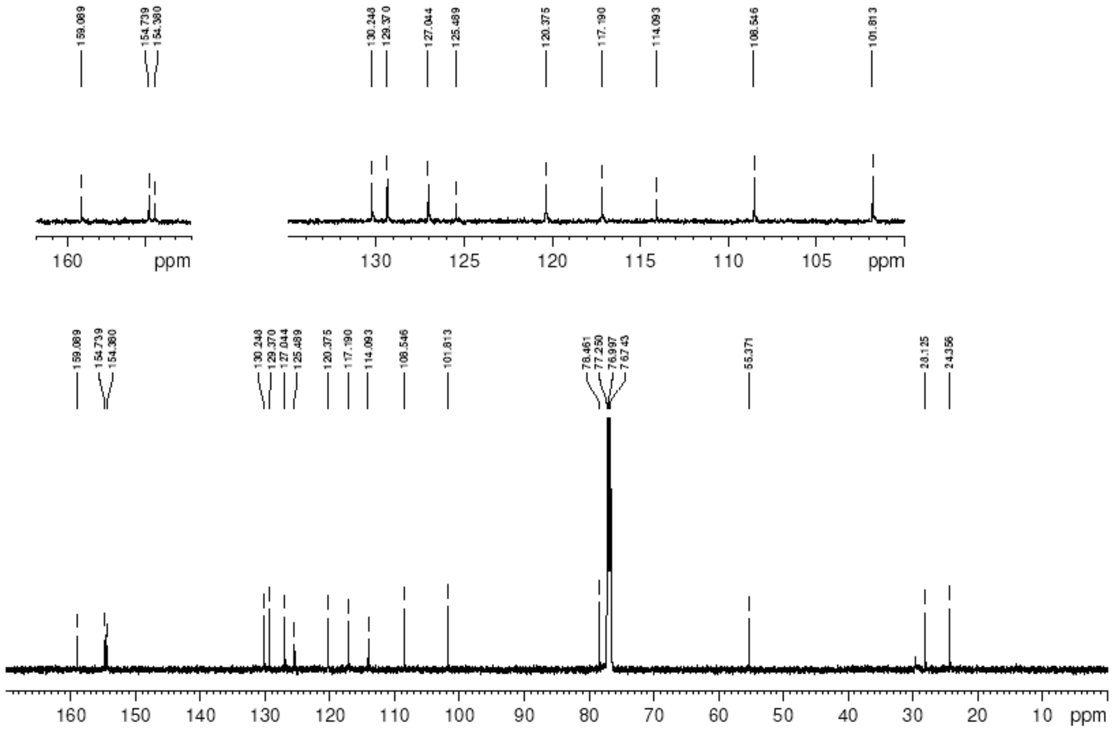

Figure S21. The ^{13}C-NMR spectrum of (2*S*)-2'-hydroxy-7-methoxyflavan (**3**; 125 MHz, $CDCl_3$)

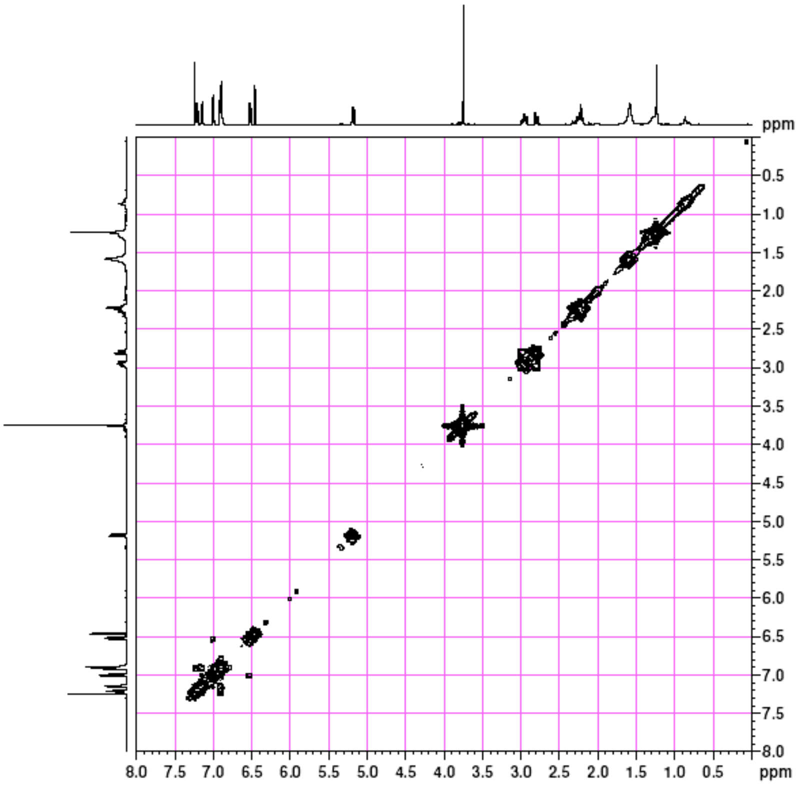

Figure S22. COSY spectrum of (2*S*)-2'-hydroxy-7-methoxyflavan (**3**)

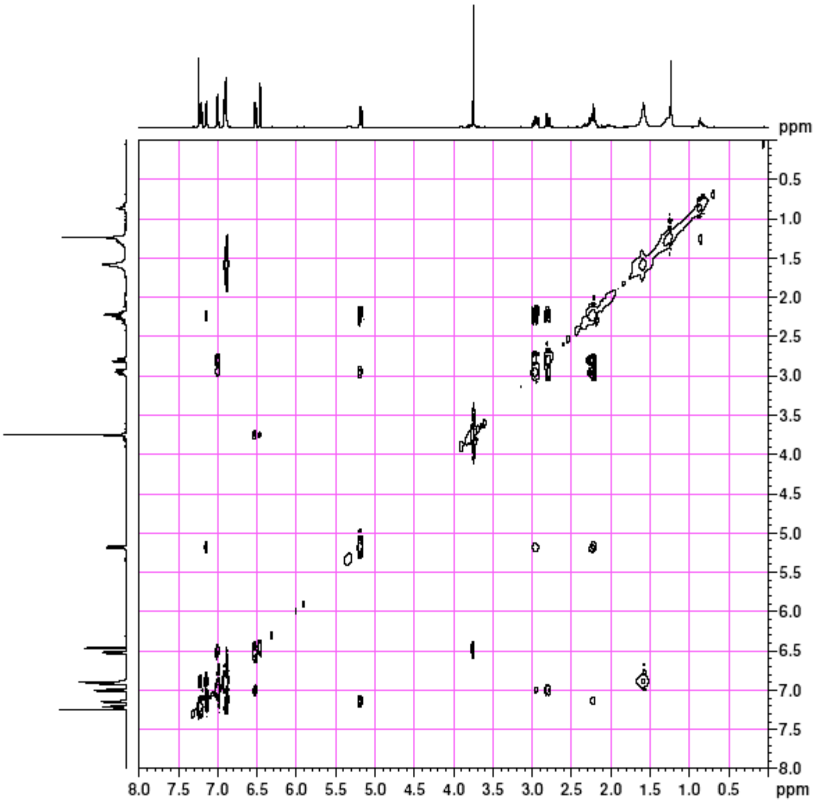

Figure S23. NOESY spectrum of (2*S*)-2'-hydroxy-7-methoxyflavan (**3**)

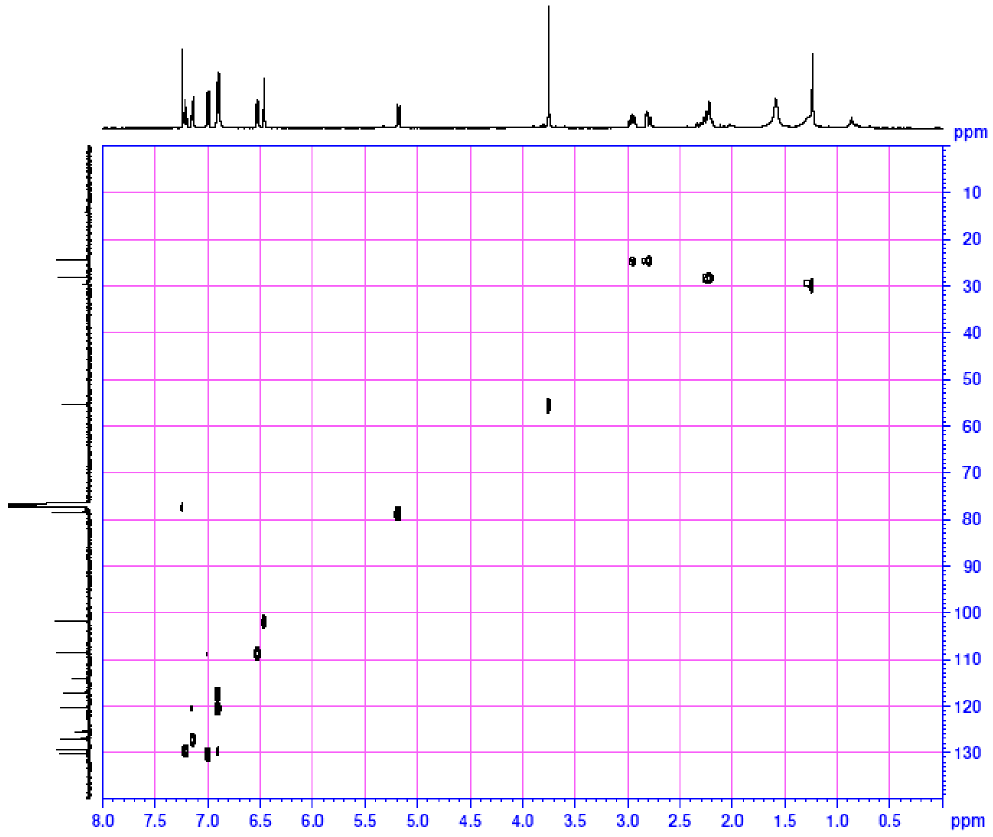

Figure S24. HMQC spectrum of (2*S*)-2'-hydroxy-7-methoxyflavan (**3**)

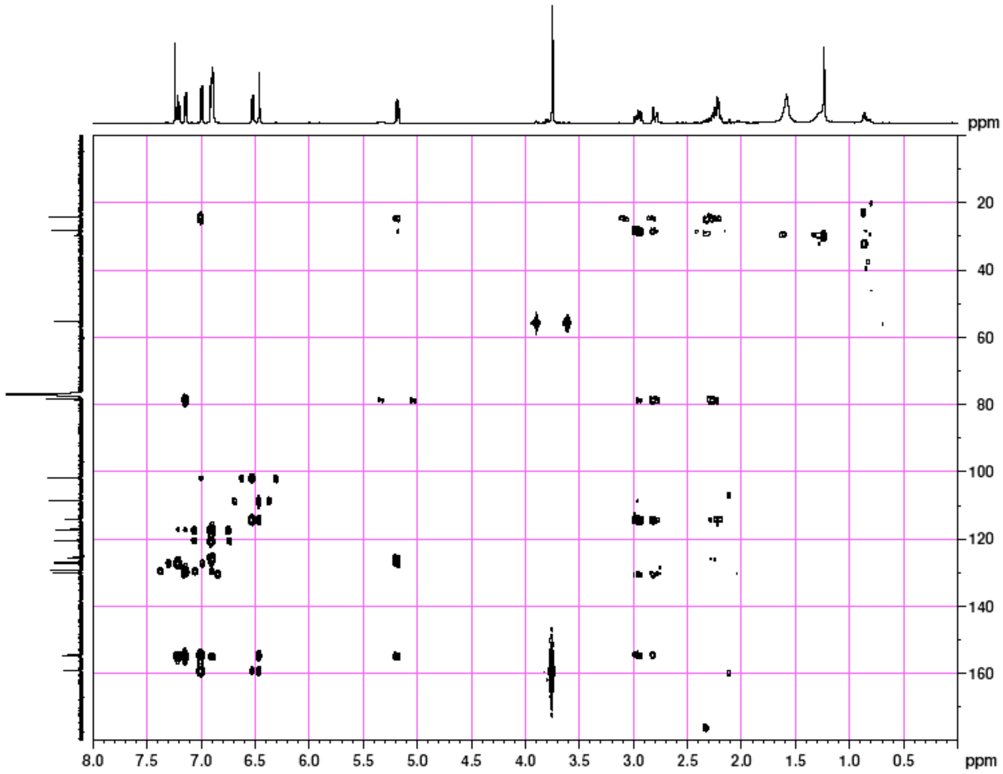

Figure S25. HMBC spectrum of (2*S*)-2'-hydroxy-7-methoxyflavan (**3**)

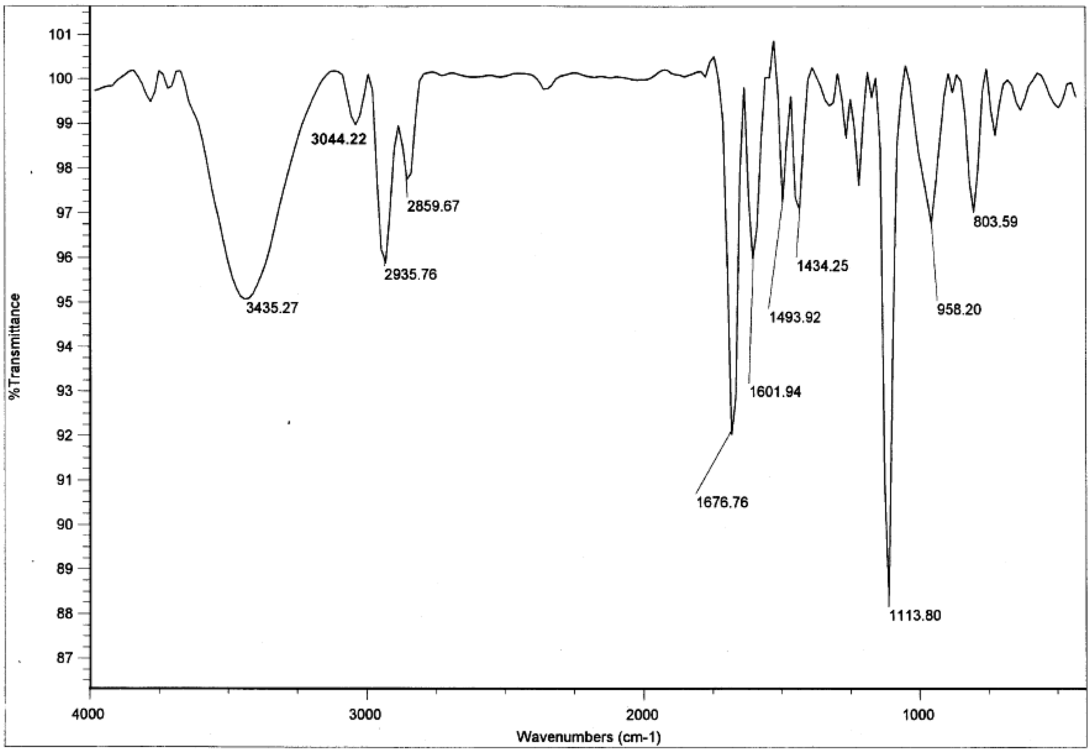

Figure S26. IR spectrum of 4-hydroxy-4-(7-methoxy-8-methylchroman-2-yl)-cyclohex-2-enone (**4**)

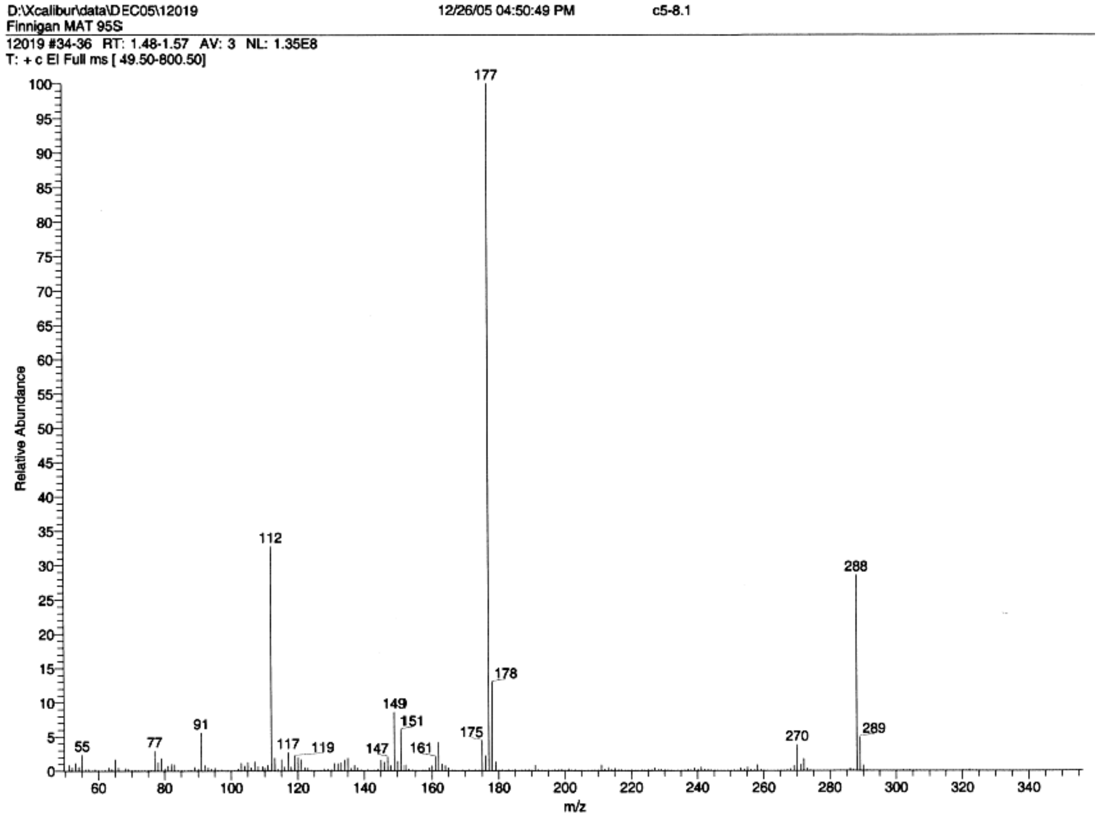

Figure S27. EIMS spectrum of 4-hydroxy-4-(7-methoxy-8-methylchroman-2-yl)-cyclohex-2-enone (**4**)

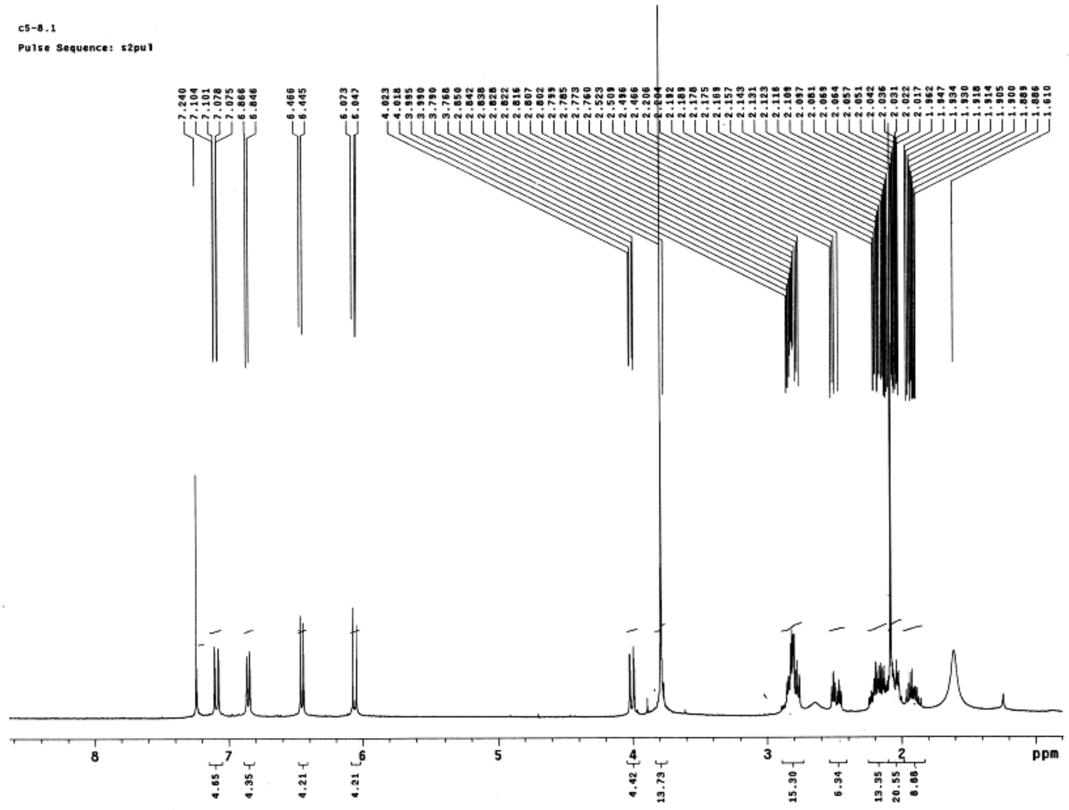

Figure S28. The ^{1}H-NMR spectrum of 4-hydroxy-4-(7-methoxy-8-methylchroman-2-yl)-cyclohex-2-enone (**4**; 400 MHz, $CDCl_3$)

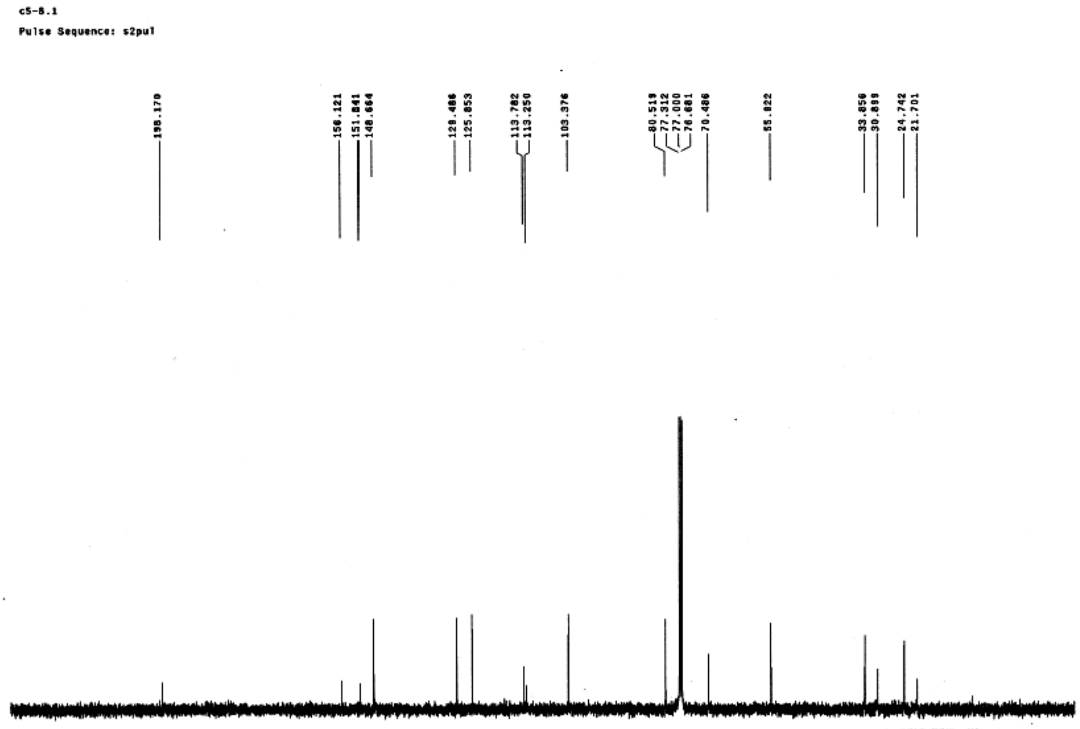

Figure S29. The ^{13}C-NMR spectrum of 4-hydroxy-4-(7-methoxy-8-methylchroman-2-yl)-cyclohex-2-enone (**4**; 100 MHz, $CDCl_3$)

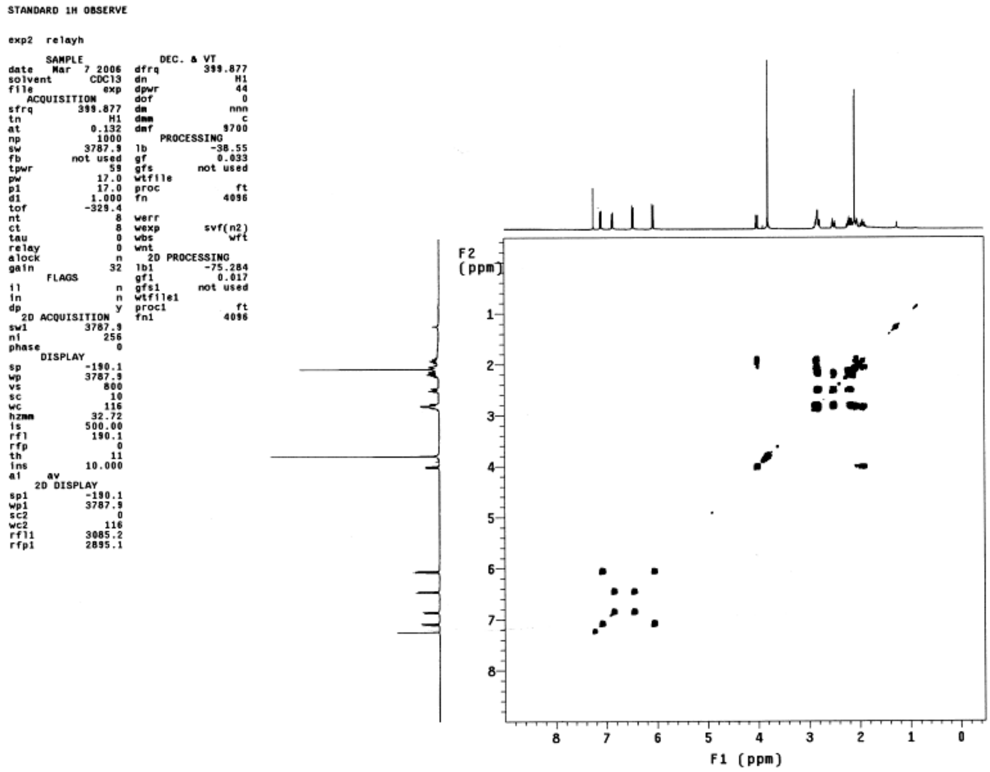

Figure S30. COSY spectrum of 4-hydroxy-4-(7-methoxy-8-methylchroman-2-yl)-cyclohex-2-enone (**4**)

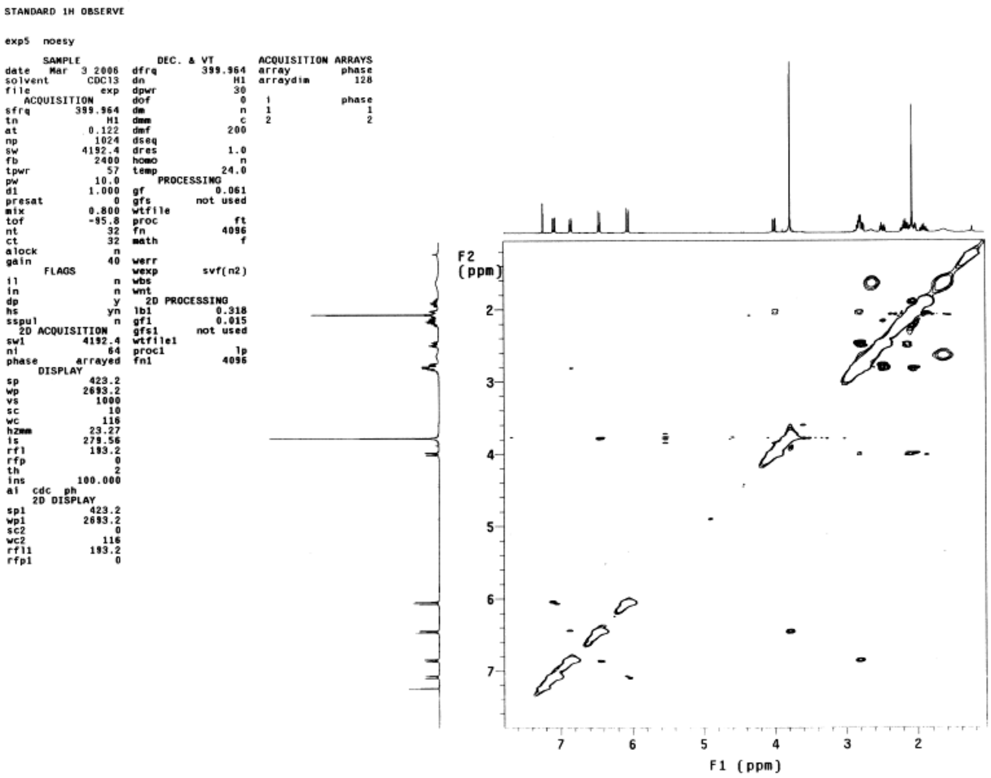

Figure S31. NOESY spectrum of 4-hydroxy-4-(7-methoxy-8-methylchroman-2-yl)-cyclohex-2-enone (**4**)

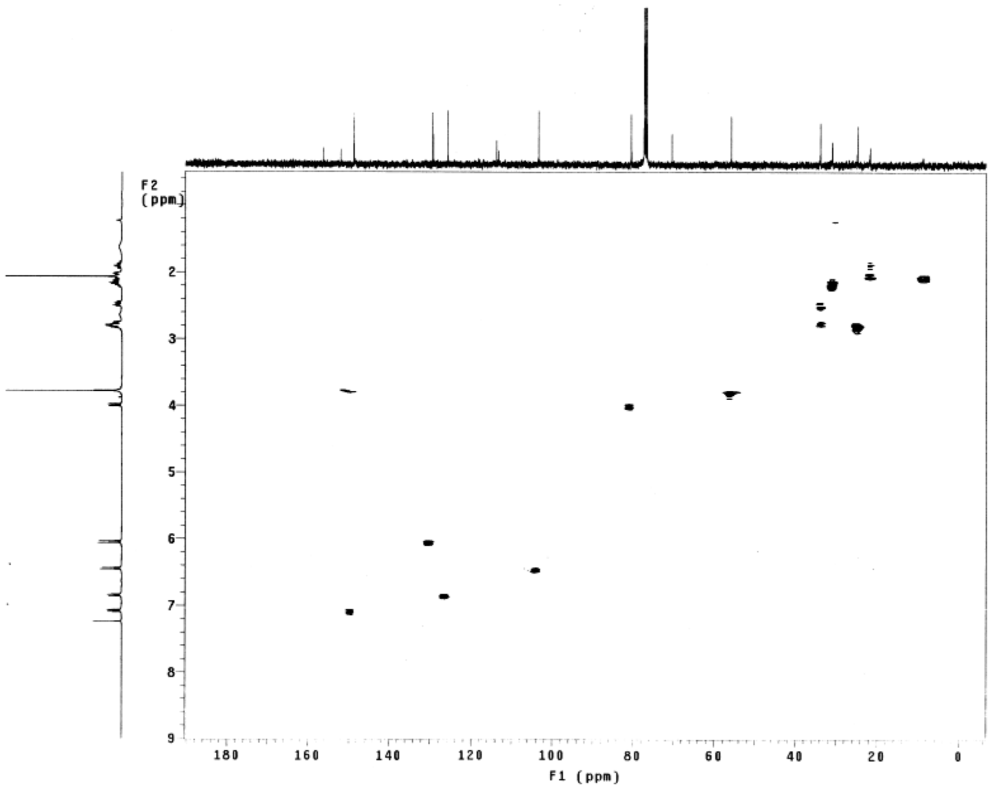

Figure S32. HMQC spectrum of 4-hydroxy-4-(7-methoxy-8-methylchroman-2-yl)-cyclohex-2-enone (**4**)

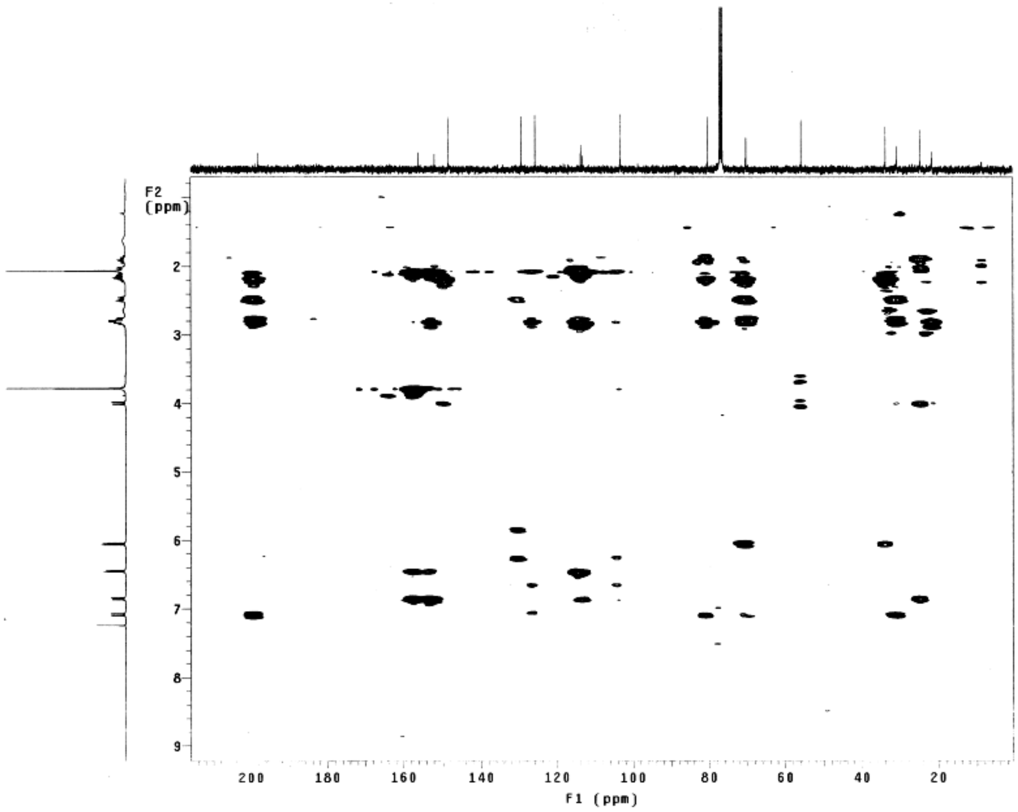

Figure S33. HMBC spectrum of 4-hydroxy-4-(7-methoxy-8-methylchroman-2-yl)-cyclohex-2-enone (**4**)
